# *Chrysosporium articulatum* mimicking *Trichophyton* spp. infection in a cat: a case presentation and literature review

**DOI:** 10.1186/s12917-024-04185-7

**Published:** 2024-08-10

**Authors:** Magdalena Kizerwetter-Świda, Iwona Bąk, Małgorzata Justyna Biegańska, Kourou Dembele, Dorota Chrobak-Chmiel

**Affiliations:** 1 Department of Preclinical Sciences, Institute of Veterinary Medicine, Warsaw University of Life Sciences-SGGW, Ciszewskiego Str. 8, Warsaw, 02-786 Poland; 2 Department of Small Animal Diseases and Clinic, Institute of Veterinary Medicine, Warsaw University of Life Sciences-SGGW, Nowoursynowska 159c, Warsaw, 02-776 Poland

**Keywords:** Dermatophytosis, *Chrysosporium articulatum*, *Trichophyton* spp., Misidentification

## Abstract

**Background:**

Dermatophytosis is a common skin infection of cats and many other animals. A reliable diagnosis is crucial because of the zoonotic potential of dermatophytes. The routine mycological diagnostic procedures for dermatophytosis are widely known, but in the case of some isolates, identification based on phenotypic characteristics may be incorrect. Infections caused by *Chrysosporium* spp. are usually described in reptiles, but in other animals they are uncommon.

**Case presentation:**

This study presents a description of a cat with dermatological lesions, that was mistakenly diagnosed with *Trichophyton* spp. dermatophytosis. Clinical material for mycological examination was collected from alopecic areas on the back of the neck, the ventral abdomen, and the hindlimbs. The initial identification based on phenotypic properties indicated *Trichophyton* spp. The result of the MALDI-ToF MS allowed the exclusion of the *Trichophyton* genus. Ultimately, the correct identification as *Chrysosporium articulatum* was obtained based on the sequencing of ribosomal genes.

**Conclusions:**

Interpretation of the results of the mycological examination of samples collected from animals’ skin or hair shafts is always challenging. Thus, careful consideration of the primary cause of the clinical lesions observed on the skin is mandatory, and the culture results are worth supporting by molecular methods.

## Background

Dermatophytosis is a common fungal infection in veterinary and human medicine. Dermatophytes are filamentous fungi that may cause superficial infections of keratinized tissues such as skin (stratum corneum of the epidermis), hairs and claws in different animal species, including dogs and cats. The vast majority of dermatophytoses in pets are caused by *Micropsorum* spp. and *Trichophyton* spp. [1–4]. The pathogenicity of these fungi is related to their ability to degrade keratin found in superficial tissues, typically viable tissues are rather not invaded. However, sporadic invasive infections have been reported in immunocompromised or elderly human patients [5]. Dermatophytes belong to a group of keratinophilic and keratinolytic fungi. In addition, many keratinophilic environmental fungal species can use pre-digested keratinaceous debris or by-products of keratin degradation. These are: *Chrysosporium* spp., *Psuedogymnoascus* spp., *Geomyces* spp., *Pectinotrichum* spp., *Renispora* spp. and others. In general, these non-dermatophytes keratinolytic fungi are saprophytes, engaged in the decomposition of keratinized residues in the soil. However, *Chrysposporium* spp. strains with kertinolytic properties have been described, with positive results in hair perforation test [6, 7].

*Chrysosporium* genus is classified in the family *Onygenaceae*, *Onygenales* order, *Eurotiomycetes* class and *Ascomycota* phylum. This genus includes about 100 species [8], commonly found in the environment, soil, and water sediments, but also on the skin and hairs of animals and humans. The taxonomical classification is often based on the fungal morphology. However, when sexual states and macroconidia are not present, the microconidia-producing fungi are clustered in polyphyletic genera, such as the genus *Chrysosporium*. Recent research results based on genetic properties have allowed the updating of the *Chryspsporium* spp. taxonomy [9]. Moreover, Kendemir et al. (2022) have shown 100% ITS sequence identity in *C. articulatum* UAMH 4320 with *Aphanoascus reticulisporus* [10]. Colonies formed by *Chrysosporium* are white or pale with septate hyphae producing pyriform or obovate to ellipsoidal microconidia [6]. The appearance of these powdery colonies as well as micromorphology resembles some dermatophytes, e.g. *Trichophyton mentagrophytes* [11]. Fungi classified in the genus *Chrysosporium* are regarded as non-pathogenic. However, there has been an increasing number of infections caused by these fungi in recent years. Most of the documented cases involve immunocompromised humans [12, 13]. Infections of this etiology also occur in animals, mainly in reptiles, most often as cases of dermatitis, but also as life-treating infections [14, 15]. *Chrysosporium tropicum* was described as a causative agent for dermatomycosis in chickens [16]. Additionally, *Chrysosporium* spp. is often isolated from feathers [17].

The clinical manifestations of dermatophytosis in cats are variable and related to the dermatophyte species involved [18]. Typically, single or several alopecic areas with scaling, crusting and erythema are observed. However, other clinical presentations are also possible, like a matted coat, seborrhea, miliary dermatitis, the presence of pustules, papules, macules, nodules, hyperpigmentation, kerions, and onychomycosis. Infected animals may show symptoms of pruritus. The variable clinical appearance of dermatophytosis can be explained by differences in the composition and structure of keratin, the specificity of enzymes produced by particular fungi, and the defence mechanisms of host organisms [18, 19]. Moreover, any other dermatoses may cause similar clinical manifestations. Thus, differential diagnosis including, among others food allergy, hormonal disorders, atopic dermatitis, autoimmune dermatoses, bacterial dermatitis, or infestation with skin parasites should always be performed. Hence, the diagnostic procedures must be accurate and carried out step-by-step. Apart from mycological examination, the results of additional tests such as parasitic, bacteriological, histopathology of biopsy material and allergy tests should also be performed [19]. Of note, the reliable diagnosis of dermatophytosis in dogs and cats is also essential because of the zoonotic potential of most of the species isolated from pets [20]. Moreover, cats may be asymptomatic carriers of *M. canis* or they may have a subclinical infection, which further complicates the diagnosis [3].

In this study we present a case of a cat with dermatological lesions, initially diagnosed with *Trichophyton* spp. infection. Ultimately, the cultured fungi were identified by sequencing and matrix-assisted laser desorption ionization-time of flight mass spectrometry method (MALDI-ToF MS) as *C. articulatum*, which is usually regarded as a non-pathogenic fungus. Moreover, we present a review of diagnostic procedures used in dermatophyte identification and the literature data on infections caused by *Chrysosporium* spp.

## Case presentation

**Fig. 1:**
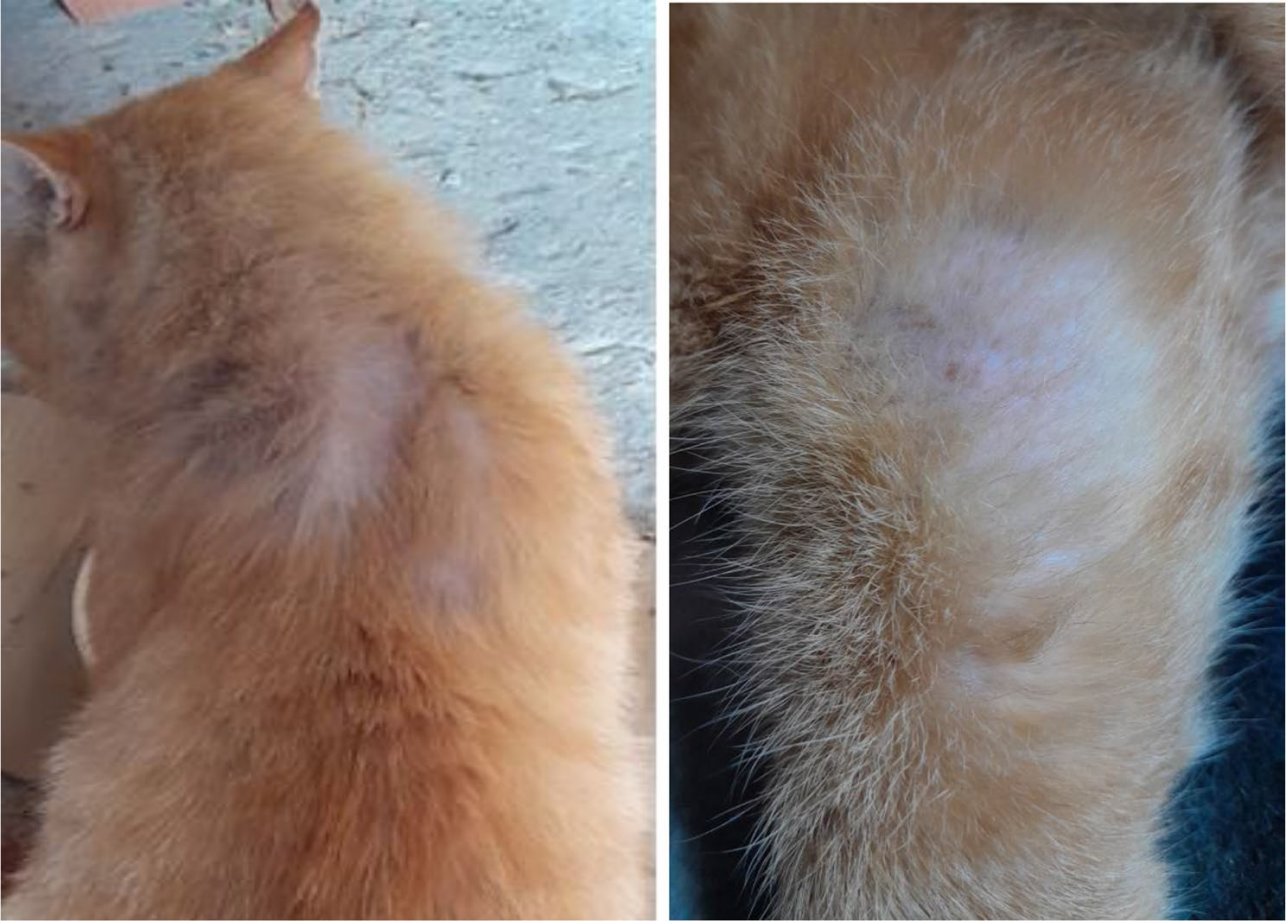
Pruritic self-inflicted alopecic areas on the back of the neck (left) and the hindlimb (right)

A 7-year-old an outdoor, neutralized male European shorthair cat weighing 6 kg showing dermatological lesions was admitted to the Small Animal Clinic at the Institute of Veterinary Medicine, Warsaw University of Life Sciences. Clinical findings included: intense pruritus and alopecia on the back of the neck, on the ventral abdomen, and the hindlimbs (Fig. 1). At the visit, flea dermatitis was excluded. Wood’s lamp examination was performed, and no fluorescence was observed. The cat was diagnosed with dermatitis miliaris. To reduce intense itching, the cat was treated with dexafort (0,9 mg i.m.). Plucked hairs and scraped scales were collected for mycological examination.

**Fig. 2:**
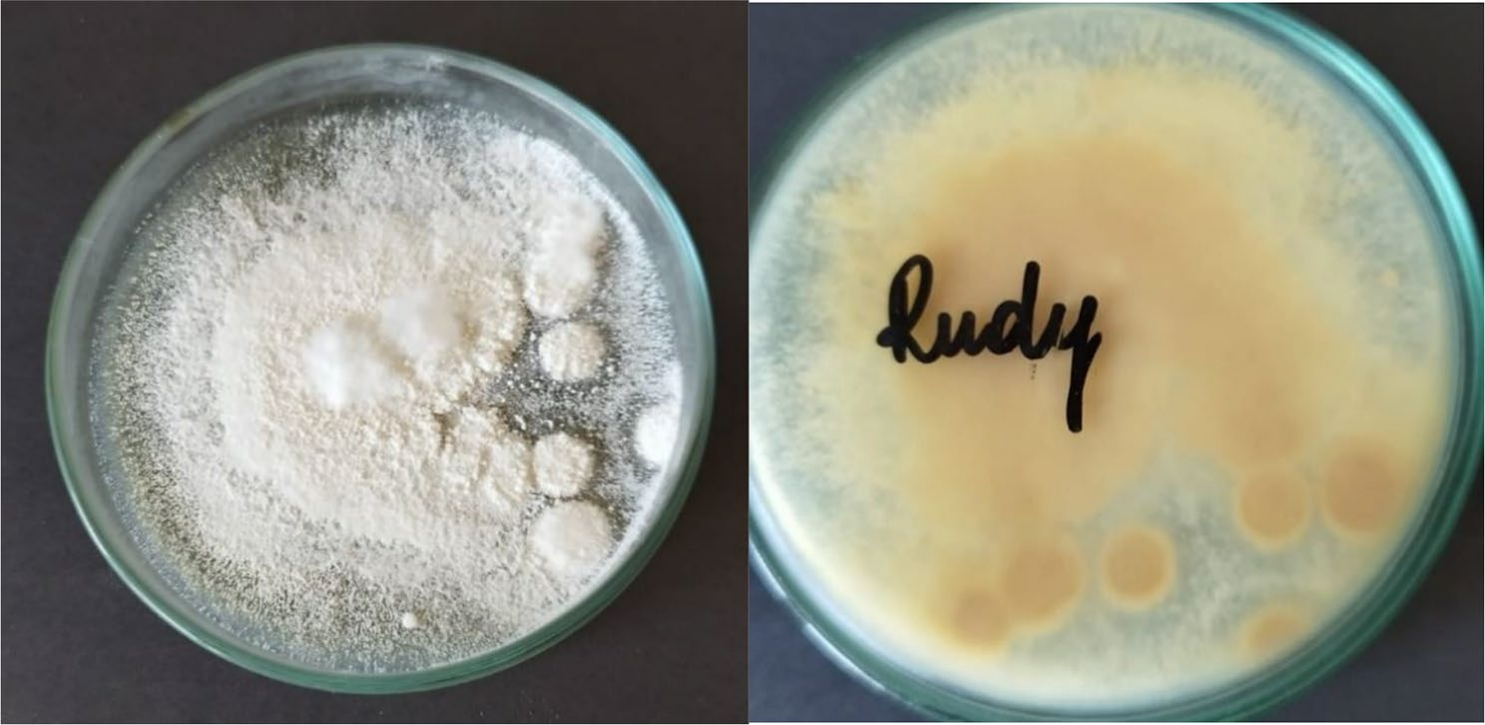
Colony morphology on SDA medium supplemented with chloramphenicol and cycloheximide (front and back of the plate) - flat, white colonies, with a powdery surface on the front and pale brown on the reverse

**Fig. 3:**
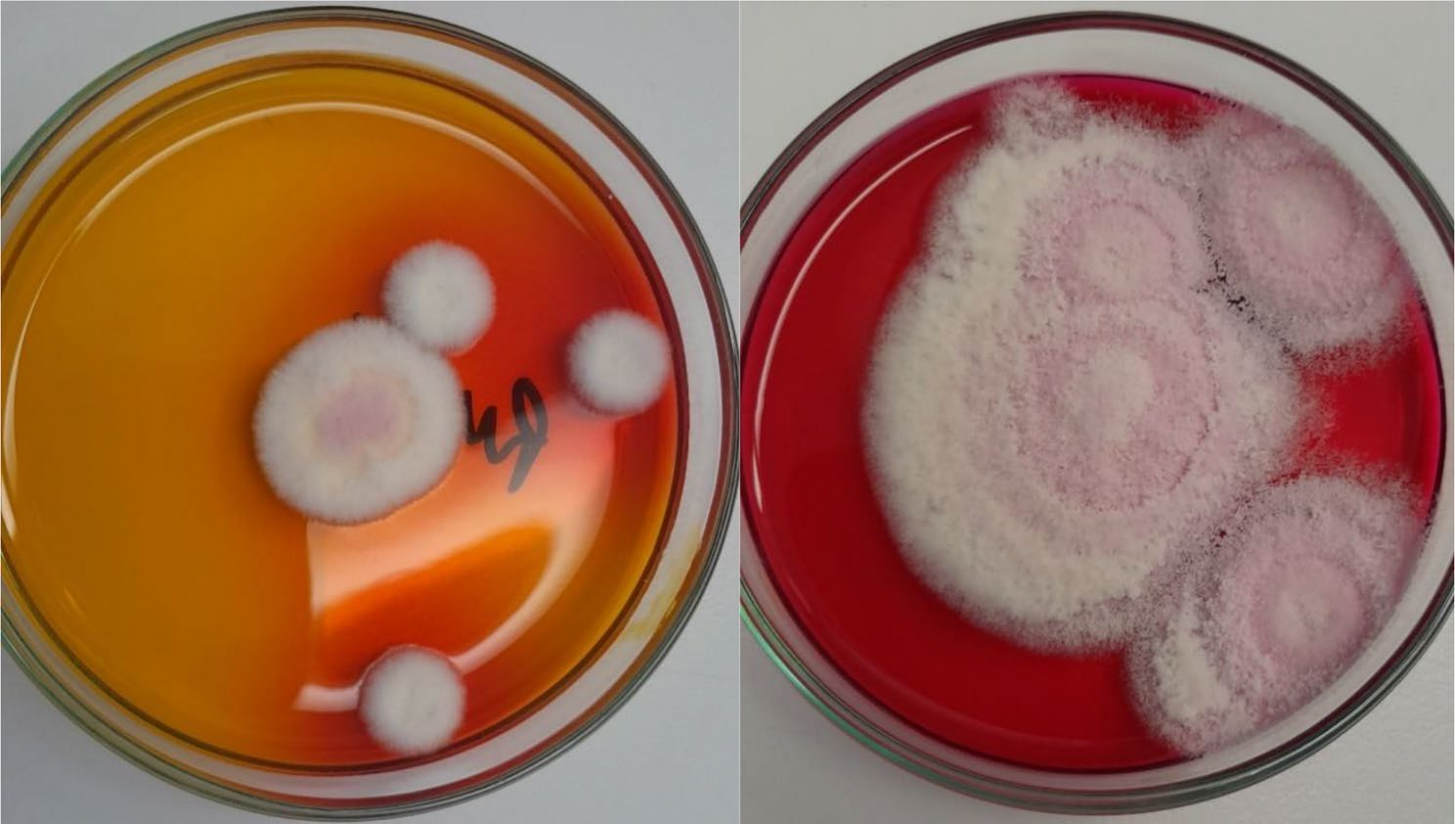
Colony morphology on DTM medium - colour change from yellow to red (five days of incubation on the left and four weeks of incubation on the right)

**Fig. 4:**
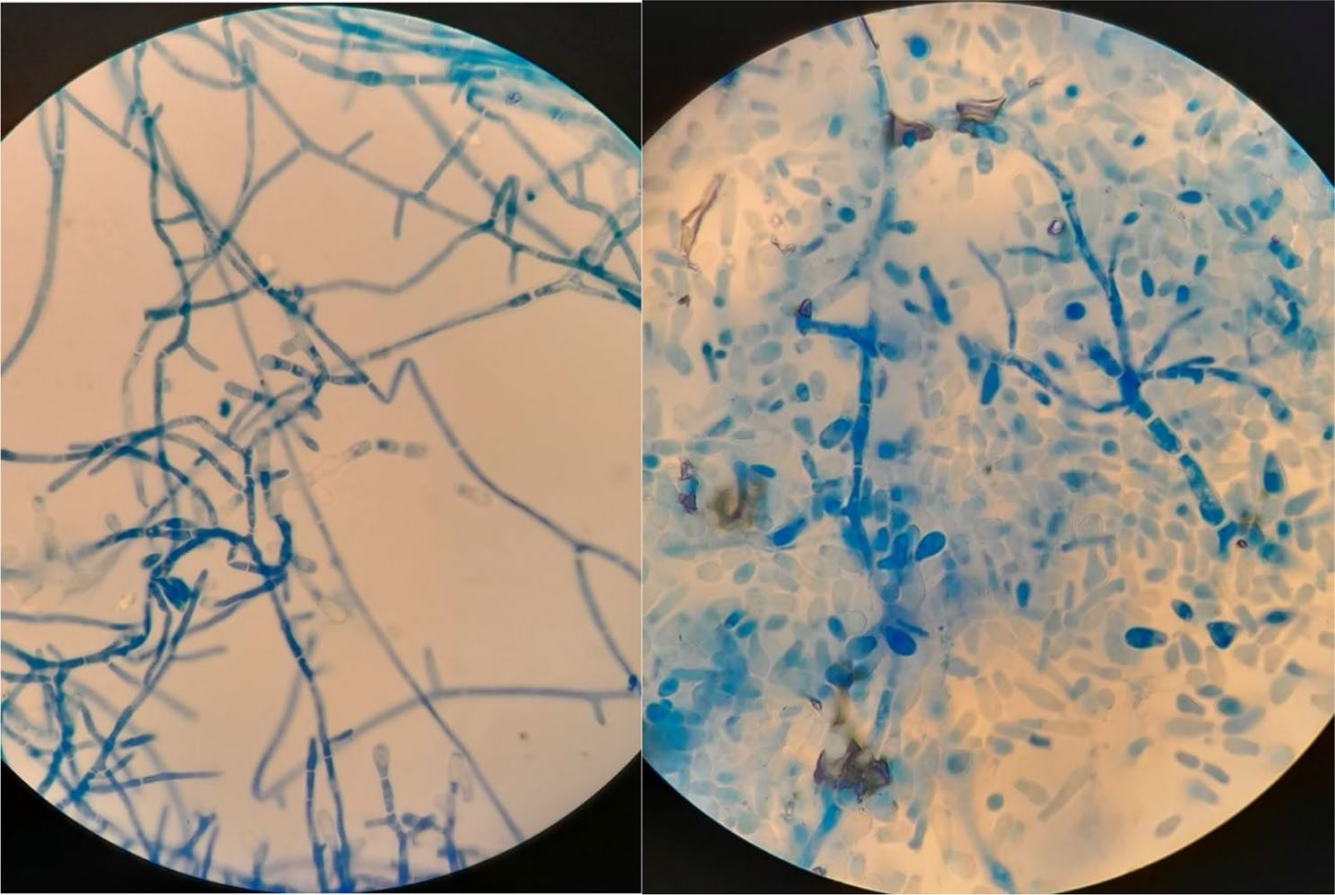
Morphology of septate hyphae and microconidia – light microscope examination under 400x magnification with lactophenol cotton blue staining

Direct microscopic examination of collected hairs and scales was performed with KOH, but wet-mounts failed to detect any spores or other fungal elements in both examined samples. Sabouraud dextrose agar (SDA), Sabouraud dextrose agar supplemented with 0.05% cycloheximide and 0.005% chloramphenicol, and dermatophyte test medium (DTM) were used for fungal culture. All plates were incubated aerobically, at 25 °C for four weeks. The colonies appeared on SDA and DTM medium after five days of incubation. Colonies were flat, white in colour, with a powdery surface (Fig. 2). DTM medium turned red, as is observed when dermatophytes grow. Colony morphology resembled colonies of *Trichophyton* spp. (Fig. 3). The isolate was examined for microscopic morphology using lactophenol cotton blue staining. Conidia were smooth and thin-walled, pyriform, one-celled, and sessile, usually on side branches or at the ends of long narrow stalks (Fig. 4). Additionally, a hair perforation test was performed following standard mycological procedures, and no keratinolysis was noted. The isolate was identified based on the colony morphology on SDA, DTM medium and micromorphology as *Trichophyton* spp. Thus, topical and systematic antifungal therapy was prescribed.

The fungal isolate was further identified using MALDI Biotyper (Bruker Daltonics, Billerica, MA, USA) according to the manufacturer’s instruction at the Jagiellonian Centre of Innovation (Kraków, Poland). The identification of our isolate with the MALDI-ToF MS method revealed *Chrysosporium keratinophilum* with a score value of 2.11. The identification score ranging 2.00–3.00 was considered as a high-confidence identification to the species level.

Ultimately, molecular biology methods were used for identification. Genomic DNA was extracted from five-day-old colonies using the method described by Brillowska-Dabrowska et al. [21]. Briefly, a fragment of a colony was mixed with 100 µl of extraction buffer (60 mM sodium bicarbonate, 250 mM potassium chloride and 50 mM Tris, pH 9.5, Sigma Aldrich) and incubated at 95 °C for 10 min. Then, 100 µl of 2% bovine serum albumin was added and after vigorous vortexing for 5 s, the obtained solution was used for PCR. Amplification of the internal transcribed sequence (ITS) region of ribosomal RNA was used with conserved primers ITS4 and ITS5 described by White et at. [22], with the following thermal-cycling conditions: initial denaturation for 3 min at 94 °C, followed by 35 cycles of 30 s at 94 °C, 30 s at 50 °C, 45 s of at 72 °C, and final elongation for 6 min. The obtained product was verified by agarose gel electrophoresis and subjected to sequencing with the same primers. Finally, the sequence was analyzed with BLAST software using the National Center for Biotechnology Information (NCBI) database. GenBank BLAST analysis of the obtained sequence of the internal transcribed sequence region of ribosomal RNA indicated 99.27% identity to a sequence of *Chrysosporium articulatum* deposited in the NCBI database.

Finally, the isolate obtained from a cat was recognized as *C. articulatum*, which was considered an environmental isolate contaminating the fur. Based on the verified identification dermatophytosis was ultimately excluded, allowing to avoid unnecessary implementation of antifungal therapy to the patient. The final diagnosis was a food allergy, with the recommendation of an elimination diet. After four weeks, a follow-up visit took place, during which the veterinarian confirmed that the cat’s condition improved, in alopecic areas, fur started to regrow and the itching had stopped. During the follow-up visit, hair samples were collected for mycological culture, which gave a negative result.

## Discussion

Veterinary mycological diagnostics encounter certain difficulties in identifying unusual, less frequently isolated species. The positive fungal culture results in invasive infections or disseminated cutaneous infections and does not pose any problems in interpretation because the clinical samples are collected from tissues and should not contain any fungal elements, including saprophytes. The cultivation of fungi commonly considered environmental saprophytes from superficial skin lesions is more challenging in interpretation. It may be difficult to assess whether these fungi caused the infection (in some immunocompromised patients) or whether they were cultivated accidentally. Moreover, in some cases, the differentiation of dermatophytes and other non-dermatophytic fungi may be more demanding than it seems. Incorrect identification of pathogenic fungi as saprophytes may result in the omitting of necessary antifungal therapy despite the medical indications. Alternatively, therapy may be introduced for patients that do not require such treatment, because only environmental saprophytic fungi were cultured from samples collected superficially. The treatment of dermatophytosis in dogs and cats may be topical or quite often requires systemic administration of antifungals [23]. Topical therapy is used to minimize disease transmission and environmental contamination, while systemic antifungal therapy eradicates the infection within the hair follicle [24]. Treatment of dermatophytosis may be associated with side effects, such as liver toxicity or vasculitis, and it may lead to an increase in fungal resistance. Unnecessary antifungal treatment, which is usually long-term, causes an imbalance in natural microbiota.

Fungi classified in the genus *Chrysosporium* are regarded as non-pathogenic, non-dermatophyte keratinolytic fungi. Recently, the number of cases of human infections caused by *Chrysosporium* spp. described in the literature is increasing, especially in immunocompromised human patients. *Chrysosporium zonatum* and *Chrysosporium tropicanum* are most commonly reported [25]. The clinical presentation includes respiratory allergic reactions, pulmonary invasive infections and skin infections. There is only one documented case of *Chrysosporium articulatum* invasive pulmonary infection in human, 16-year-old man diagnosed with lymphoblastic leukemia [12].

In veterinary medicine infections caused by *Chrysosporium* spp. are rarely described, and mostly are reported in reptiles. In recent years, *Chrysosporium* anamorph of *Nannizziopsis vriesii* (CANV) has become the leading fungal agent of dermatitis in reptiles. The lesions initially involve the skin, and the presence of hyperkeratosis, necrosis, vesicles, crusts, and ulceration may be observed. Progress to fatal systemic disease often occurs [14, 15].

We have gathered here five literature reports concerning *Chrysosporium* spp. infections in dogs and cats. Of note, publications describing the isolation of these fungi from before 1990 have been omitted due to the unreliable identification methods used at that time. The first is a review study concerning 157 cases of disseminated canine mould infections demonstrated that the majority (59,3%) was caused by *Aspergillus* spp. *Chrysosporium* spp. was identified as the etiological agent only in two cases, which corresponds to only 1,3% of incidence [26]. One of the publications included in the review mentioned above was a case report concerning disseminated infection in German shepherd dog in Australia. Fungal hyphae were observed in needle aspirates of the iliac lymph nodes and spleen. The fungal culture from these materials was positive and was diagnosed as *Chrysosporium* spp. [27]. An earlier publication also from Australia described disseminated opportunistic fungal infections among 10 dogs, of which, in one case, *Chrysosporium* spp. was found to be the etiological agent [28]. In another review study describing fungal keratitis in 11 dogs, the presence of *Chrysosporium* spp. was confirmed in one patient [29]. Moreover, the literature provides one description of superficial skin lesions in two Persian cats and their owner caused by *Chrysosporium* spp. These two cats lived in the same household. Moreover, *Chrysosporium* spp. was also isolated from its owner, who was undergoing chemotherapy for mammary cancer. Fungal culture from hairs and skin scrapings revealed the presence of *Chrysosporium* spp. in both cats. Unfortunately, the authors did not verify the identification with molecular biology methods, however, effective antifungal treatment proved, that the isolated fungi were the etiological agent involved in the observed clinical changes [30]. Additionally, in 2011 Pin et al. described well-documented onychomycosis caused by *C. keratinophilum* in seven captive Bennett’s wallabies [31].

Diverse fungal species may occur on the skin and hairs of cats, which may be either pathogens or contaminating saprophytes. Thus, veterinary mycological diagnostics encounter dilemmas, such as contamination of superficial clinical samples by saprophytic fungi, which is most probable when the samples of hair, skin scrapings or claws are collected. *Chrysosporium* spp. is one of many saprophytic fungi that can contaminate the animal’s haircoat or skin and thus contribute to the contamination of clinical samples. *Chrysosporium* spp. has been most commonly isolated (25%) from healthy dogs and cats in Mexico [32]. This creates a challenge for veterinary laboratory diagnostics because *Chrysosporium* spp. shows similar characteristics to dermatophytes [7]. These fungi may have macromorphology and micromorphology similar to some *Trichophyton* spp., thus may be easily misidentified. Additionally, *Chrysposporium* spp. can grow on the DTM agar, causing pH change and redness of the medium while showing morphological characteristics corresponding to dermatophytes [30]. Furthermore, a positive hair perforation test was observed for *Chrysosporium* species. isolated from the environment, confirming their keratinolytic properties. Mitola et al. have described positive results of a hair perforation test for *Chryspsporium georgii*, *Chrysosporium keratinophilum*, and *Chrysosporium lucknowense* isolates obtained from environmental samples [7]. Likewise, Gurung et al. observed keratinolytic activity in soil isolates identified as *Chrysosprium indicum* and *Chrysosporium fluviale* [6].

A common opinion is that dermatophytes may be easily discriminated with DTM agar plate. However, literature data indicate that other fungi can also produce a positive reaction in this medium. These include *Chrysosporium* spp., as confirmed by Dokuzeylul et al. [30] and Jang et al. [33]. Jang et al. (2007) found that 63% of moulds isolated from dogs produced colour changes to red on DTM medium, including *Chrysosporium*, as well as some isolates of *Aspergillus*, *Penicillium* and others. Thus, as mentioned before, the color change of DTM agar is not sufficient to confirm the presence of dermatophytes.

The identification of our isolate with MALDI-ToF MS showed *Chrysosporium keratinophilum* with a high score value of 2.11. However, the sequencing of ribosomal genes indicated *Chrysosporium articulatum*. While performing MALDI-ToF MS analyses, the manufacturer’s Brucker database included protein spectra from only two species of this genus (*C. keratinophilum* and *Chrysosporium shanxiense*). Therefore, we were unable to obtain correct species identification with this method. Nevertheless, the high score value of *C. keratinophilum* allows us to exclude *Trichophyton* spp. Similar difficulties in the identification of filamentous fungi were described by Normand et al. [34] and Wilkendorf et al. [35]. The explanation for this situation is that proteomic profiles of unusual, saprophytic, filamentous fungi are currently not included in available databases, also indicating the need to expand and update these databases.

## Conclusion

Our report describes a case of a cat with dermatological lesions initially misdiagnosed as dermatophytosis caused by *Trichophyton* spp. The initial identification of DTM-positive isolate as *Trichophyton* spp. was confirmed by colony morphology on Sabouraud agar as well as its micromorphology. Nevertheless, correct identification to the species level was obtained after sequencing of ribosomal genes. The identification using the MALDI-ToF MS technique was not possible because the available database does not include this species. Although this method allowed for the recognition of the genus *Chrysosporium*. Results presented in this study indicate that interpretation of the results of the mycological examination in all cases of culturing saprophytic fungi, growing from superficial samples is always challenging. Thus, careful consideration of the primary causative agent of the clinical lesions observed on the skin is mandatory. Moreover, DTM medium should be used only as a screening method, and the identification of DTM-positive isolates as dermatophytes must be confirmed by other tests.

## Data Availability

The dataset generated and analyzed during the current study is available in the NCBI GenBank repository, under the accession number: PP758650.

## Abbreviations

MALDI-ToF MS: Matrix assisted laser desorption ionization-time of flight mass spectrometry
SDA: Sabouraud dextrose agar (SDA); DTM - Dermatophyte test medium
